# Does dexmedetomidine infusion reduce the postoperative analgesic need in lumbar disc surgery?

**DOI:** 10.55730/1300-0144.5991

**Published:** 2025-03-24

**Authors:** Sibel ÇATALCA, Özlem ÖZMETE, Numan BERK, Soner ÇİVİ, Emre DURDAĞ, Caner İNCEKAŞ, Nesrin BOZDOĞAN ÖZYILKAN

**Affiliations:** 1 Department of Anesthesiology and Reanimation, Faculty of Medicine, Başkent University, Adana, Turkiye; 2 Department of Neurosurgery, Faculty of Medicine, Başkent University, Adana, Turkiye; 3 Department of Biostatistics, Faculty of Medicine, Başkent University, Ankara, Turkiye

**Keywords:** Dexmedetomidine, general anesthesia, lumbar microdiscectomy, postoperative analgesia

## Abstract

**Background/aim:**

Patients experience moderate-to-severe pain, especially in the first days after lumbar disc surgery. Poorly controlled pain in the postoperative period negatively affects patient outcomes. Dexmedetomidine is a highly selective α2 adrenoceptor agonist with demonstrated analgesic efficacy. However, conflicting results have been reported in the current literature regarding the efficacy of dexmedetomidine in this surgery. In this study, we tested the hypothesis that dexmedetomidine safely improves pain scores and reduces opioid consumption in lumbar microdiscectomy.

**Materials and methods:**

Medical records of patients who underwent lumbar microdiscectomy with general anesthesia between January 2023 and October 2023 were retrospectively reviewed. Patients who met the inclusion criteria were divided into two groups as those who did not receive dexmedetomidine infusion (Group A) and those who received dexmedetomidine infusion (Group B). Patients in Group B received a loading dose of 1 μg/kg dexmedetomidine followed by a maintenance infusion of 0.5 μg/kg/h. The primary outcome of our study was postoperative fentanyl consumption at the 24^th^ h. Secondary outcomes of our study included need for fentanyl in the recovery unit, postoperative pain scores at the 2^nd^, 6^th^, 12^th^, and 24^th^ h and fentanyl consumption at the 2^nd^, 6^th^, and 12^th^ h and perioperative complications.

**Results:**

A total of 68 patients were included in our study, 34 patients in each group. The number of patients requiring fentanyl in the recovery unit and the dose of fentanyl administered were similar in both groups (p = 0.223 and p = 0.373, respectively). There was no statistical difference in the pain scores, opioid consumption, and perioperative complications at the 2^nd^, 6^th^, 12^th^, and 24^th^ h after surgery in patients receiving dexmedetomidine compared to the control group (p > 0.05).

**Conclusion:**

Intraoperative dexmedetomidine infusion did not reduce postoperative pain intensity and opioid consumption in patients undergoing lumbar microdiscectomy under general anesthesia.

## 1. Introduction

Low back pain is a common symptom in the general population. In 2020, 619 million individuals worldwide reported having low back pain. This number is predicted to reach 843 million by 2050 [1]. Lumbar disc herniation (LDH) is an important cause of low back pain [2]. Lumbar discectomy (LD) and lumbar microdiscectomy (LMD, the minimal invasive form of LD) are the surgical methods used in patients with LDH who do not respond to conservative treatment [3].

Lumbar discectomy is associated with moderate/severe pain and moderate/high-dose opioid use in the postoperative period. Acute postoperative pain caused by surgical trauma is known to affect the quality of postoperative recovery, morbidity, and mortality. Excessive and prolonged opioid use has side effects such as nausea, vomiting, respiratory depression, pruritus, opioid tolerance, and opioid-related hyperalgesia. These opioid-related side effects also negatively affect patient recovery and discharge time [4,5]. Therefore, enhanced recovery after surgery (ERAS) protocols recommend the use of multimodal analgesics that cause pain inhibition through different pathways and aim to reduce the amount of opioids administered during the perioperative process [6].

A highly selective α2 adrenoreceptor agonist, dexmedetomidine (DEX) is a centrally acting nonopioid agent that has been frequently used in clinical practice in recent years due to its antinociceptive and anxiolytic properties [7]. Many studies conducted in different surgical groups have shown that intraoperative use of DEX is effective in postoperative pain management [8,9]. However, there are also studies in the literature that do not support this result [10–12]. In addition, there are a few prospective randomized controlled studies investigating the postoperative analgesic efficacy of intraoperative infusion of DEX in the patient group undergoing spinal surgery as a primary outcome [13–15]. There is no clinical study on this subject in patients operated under general anesthesia (GA) for LMD.

The objective of this study is to evaluate the analgesic efficacy of DEX as a component of multimodal analgesia in patients undergoing LMD under GA. The hypothesis of the study is that DEX administered at a maintenance dose of 0.5 μg/kg/h after a loading dose of 1 μg/kg provides more effective analgesia.

## 2. Materials and methods

This study was approved by the Ethics Committee of Başkent University Medical and Health Sciences Research Board (number: KA 23/449, date: 16.01.2024).

### 2.1. Study population

This study was conducted by scanning the follow-up forms of patients undergoing LMD between January 2023 and October 2023. Written consents were obtained from all patients regarding the study and the analgesic treatments applied. All patients underwent elective LMD by the same neurosurgeons (SÇ, ED). The neurosurgeons performed a standard 3 ± 0.5 cm incision for each disc level.

Patients aged between 18 and 65 years, who had the American Society of Anesthesiologists Physical Status Classification (ASA) scores of I and II, and whose follow-up forms were completely filled out, were included in the study. Patients who did not undergo general anesthesia were excluded from the study. Those with a body mass index (BMI) <18 kg/m^2^ or >30 kg/m^2^, as well as noncooperative patients, were also excluded. Preoperative or postoperative neurological deficits and abnormalities in liver or kidney function tests were considered exclusion criteria. Additionally, patients with coagulopathy, advanced ischemic heart disease, or pulmonary disease were not included. Patients with advanced heart block, such as Mobitz type II or atrioventricular dissociation, and those diagnosed with uncontrolled preoperative hypertension (systolic blood pressure > 160 mmHg) were excluded. Chronic opioid users and pregnant patients were not eligible for the study. Furthermore, patients who received any analgesic other than DEX as a component of multimodal analgesia (field block, ketamine, lidocaine, magnesium, dexamethasone, morphine, pethidine, tramadol, etc.) were excluded. Patients who experienced serious arrhythmia, hypotension, or bradycardia necessitating the discontinuation of DEX infusion were not included. Finally, those who did not receive patient-controlled analgesia during the postoperative period were also excluded from the study. In our hospital, all patients were given information about the Numeric rating scale (NRS, 0: No pain, 10: The worst pain imaginable) and the use of the pain pump.

### 2.2. Study design

The data regarding the patients’ age, sex, height, weight and body mass index (BMI), comorbidities, ASA scores and smoking status were noted from the preoperative records in the hospital information system. Hemodynamic parameters at baseline, during surgical incision, at 5^th^, 10^th^, 20^th^, and 30^th^ minute (min) after surgical incision, during skin closure and during extubation were noted from the anesthesia follow-up form. Fentanyl, atropine or noradrenaline requirements during the intraoperative period, and durations of anesthesia (the time between anesthesia induction and discontinuation of sevoflurane), surgery (the time between skin incision and last skin suture), and extubation (the time between discontinuation of sevoflurane and extubation of the patient) were recorded. Complications that developed in the patients during the intraoperative period (bronchospasm, difficult hemostasis, dural injury) were also noted. Hemodynamic parameters at 0^th^, 5^th^, 10^th^, 20^th^, and 30^th^ min, complications (nausea, vomiting, shivering, pruritus, desaturation [peripheral oxygen saturation (SpO2) < 92%], Ramsey sedation scale (RSS), need for fentanyl, and time to reach Modified Aldrete score (MAS) of 9 were recorded from the follow-up forms in the recovery unit. Postoperative ward records and pain follow-up forms included fentanyl consumption and pain scores at rest and induced by movement at the 2^nd^, 6^th^, 12^th^, and 24^th^ h, need for rescue fentanyl, patient satisfaction score at the 24^th^ h using a 4-point scale (1: Not satisfied at all, 4: Very satisfied), complications (nausea, vomiting, desaturation, pruritus, shivering, urinary retention) and length of hospital stay.

### 2.3. Anesthesia management

Patients were taken to the operating room after 1 mg intravenous midazolam was administered for sedation, and an electrocardiogram, noninvasive blood pressure, and SpO2 monitoring were performed. Balanced crystalloid infusion (Isoplene-S) was started at 10 mg/kg before anesthesia induction. For induction, patients were intubated by administering 2 mg/kg propofol, 1 μg/kg fentanyl, and 0.6 mg/kg rocuronium. After intubation was confirmed, patients were placed in the prone position as appropriate. All patients were administered 1 g paracetamol and 1 g cefazolin before surgical incision. Anesthesia was maintained with age-appropriate sevoflurane MAC value (0.8–1.2) and 50%–50% oxygen-nitrous oxide mixture. Tidal volume was adjusted to 6–8 mL/kg, end-expiratory positive pressure to 5 cmH_2_O, and frequency to 30–40 mmHg end-tidal carbon dioxide. Hypothermia was prevented by using warm air heaters. In the intraoperative period, when heart rate and/or mean blood pressure increased by >20% from baseline, 0.25 μg/kg fentanyl was administered to the patients at 5-min intervals until hemodynamic stability was restored. A heart rate < 50 beats/min was defined as bradycardia, and 0.5 mg of atropine was administered. A mean blood pressure of <65 mmHg or systolic blood pressure of <90 mmHg was defined as hypotension. In this case, a fluid bolus (250 mL) was administered. When there was no response to fluid, hypotension was intervened using 10 μg noradrenaline. In case of resistant hypotension, noradrenaline was started at an infusion rate of 2 μg/min and increased to a maximum of 10 μg/kg. If hypotension persisted, DEX infusion was discontinued. All patients were administered 20 mg tenoxicam intravenously during subcutaneous fascia closure. Ten mg of metoclopramide was administered to prevent postoperative nausea and vomiting. Ten min before the last skin suture, the patients were delivered 100% O2. Sevoflurane was turned off after skin suturing was completed. Patients who complied with verbal commands and were able to open their eyes were extubated with 0.02 mg/kg atropine and 0.04 mg/kg neostigmine. Patients who were sent to the postoperative recovery unit were monitored for 30 min.

When the NRS score was >3, fentanyl (0.5 μg/kg) was administered in the recovery unit. Patients who did not have any complications were transferred to the ward after 30 min. The pain pump was initiated just before the patient was transferred to the ward. In the postoperative follow-up of the patients, 1 g paracetamol and 20 mg tenoxicam were administered intravenously twice daily in addition to the pain pump. If the NRS score was > 3 despite the paracetamol, tenoxicam, and pain pump bolus, fentanyl (0.5 μg/kg) was administered intravenously by a staff nurse as a rescue analgesic in the ward. Postoperative nausea and vomiting were treated with 3 mg granisetron in the recovery unit and 10 mg metoclopramide in the ward.

### 2.4. Study groups

The patients who met the inclusion criteria were divided into two groups: those who did not receive DEX infusion (Group A) and those who received DEX infusion (Group B). In the patient group receiving DEX, DEX was administered at a dose of 1 μg/kg for 10 min before anesthesia induction. After loading, DEX maintenance infusion was initiated at a dose of 0.5 μg/kg/h. Dexmedetomidine infusion was discontinued when skin suturing was performed. No infusion was administered to the patients in the control group.

### 2.5. Patient-controlled analgesia

Five hundred μg fentanyl (10 mL) and 3 mg granisetron (3 mL) were added to 87 mL of saline. The patient-controlled analgesia device was set as follows: Total volume: 100 mL, loading: 0 mL/h, infusion: 0 mL/h, bolus: 20 μg, lock time: 5 min, 1-h limit: 1 μg/kg.

### 2.6. Primary and secondary outcomes

The primary outcome of our study was postoperative fentanyl consumption at the 24^th^ h. The secondary outcomes of our study were the intraoperative additional fentanyl requirement, intraoperative need for atropine and noradrenaline, intraoperative hemodynamic parameters, hemodynamic parameters, RSS score, complications, additional fentanyl need and the time for MAS to reach 9 in the recovery unit. Other secondary outcomes of our study were movement-induced pain score at the 2^nd^, 6^th^, 12^th^, and 24^th^ h postoperatively, pain score at rest, fentanyl consumption at the specified times, need for rescue fentanyl, patient satisfaction at the 24^th^ h, and length of hospital stay.

### 2.7. Statistical analysis

The G*Power 3.1.9.4 (Franz Faul, Universitat Kiel, Germany) was used to calculate the minimum sample size required for our study. In this regard, the minimum sample size reached with statistics (an alpha level of 0.05, a power of 0.8 with a 95% confidence level, and an effect size of d = 0.7986762) from a previous study [15] was 26. To maximize the power of our tests, we attempted to gather data from all eligible patients.

Statistical analyses were performed using the SPSS program version 25.0. The normal distribution of the data was analyzed using the Shapiro-Wilk test. Descriptive statistics were presented as frequencies and percentages for categorical variables; means and standard deviations for continuous variables with normal distribution; and medians (minimum-maximum) for continuous variables without normal distribution or ordinal variables. Student’s t-test was used for normally distributed variables. Group comparisons of the variables that did not conform to the normal distribution were analyzed using the Mann-Whitney U test. Frequency distributions of categorical variables were analyzed with the Chi-Square test or Fisher’s exact test where appropriate. Statistical significance was accepted as p < 0.05.

## 3. Results

A total of 168 patient data sets were scanned to be included in the study. Of these patients, 5 received ketamine, 10 received tramadol, 5 received dexamethasone, 12 had uncontrolled hypertension, 8 had revision surgery, 5 had DEX infusion discontinued due to serious arrhythmia or hypotension, and 55 had incomplete records during ward follow-ups. Thus, a total of 100 patients were excluded from the study, and 68 patients were analyzed. There were 34 patients each in Group A and Group B. No significant difference was found between the groups in terms of demographic data (Table 1).

Comorbidities, anesthesia, and surgical parameters were similar in Group A and Group B (Table 2). While the additional fentanyl requirement in the intraoperative period was 43.75 ± 22.87 μg in Group A, it was 28.75 ± 16.39 μg in Group B (p = 0.212). In the intraoperative period, no significant difference was observed in hemodynamic parameters (Table 3). Bronchospasm, difficult hemostasis, or dural injury was not observed in any patient.

In the recovery unit, there was no significant difference between the groups in terms of hemodynamic parameters and RSS scores (Table 4). The number of patients requiring fentanyl in the recovery unit and the fentanyl dose administered were similar in both groups (p = 0.223 and p = 0.373, respectively). Analyses showed that there were no statistically significant differences between the groups for fentanyl consumption and NRS scores at 2^nd^, 6^th^, 12^th^, and 24^th^ h. Group A showed a nonsignificant reduction of fentanyl consumption via pain pump (251.76 μg ± 154.36 vs. 280 ± 125.82 μg, p = 0.156). The number of patients who received rescue fentanyl in the ward and the fentanyl dose were similar (p = 0.770 and p = 0.613, respectively) between the two groups (Table 5). Time for MAS 9, the number of patients who received noradrenaline and atropine perioperatively, satisfaction scores, and hospital stay were also similar between Group A and Group B (Table 6).

When the groups were examined in terms of opioid related side effects in the recovery unit, no nausea or vomiting was observed in any patient in Group A, while nausea was observed in 2 patients and vomiting in 1 patient in Group B (p = 0.493, p = 1.000, respectively). Shivering was observed in 1 patient in Group A, while no patient in Group B experienced shivering (p = 1.000). No other complications were noted in any patients. During ward follow-ups, nausea was observed in 2 patients in Group A. Vomiting, pruritus, and urinary retention were observed in 1 patient in Group A, while desaturation or shivering were not observed in any patient. In Group B, nausea was observed in 3 patients, urinary retention was observed in 1 patient, and no vomiting, pruritus, desaturation, or shivering were observed in any patient (p = 1.000).

## 4. Discussion

In this study, 1 μg/kg loading followed by 0.5 μg/kg/h DEX infusion did not improve intraoperative and postoperative fentanyl consumption and postoperative pain scores in the first 24 h in patients undergoing LMD under GA. In addition, no difference was observed in terms of intraoperative and postoperative complications.

Lumbar microdiscectomy is one of the most frequently performed surgical procedures in the appropriate patient group with low back and leg pain due to LDH [3]. Postoperative pain after LD can be moderate to severe, especially in the first days [16]. Inappropriately treated postoperative pain reduces patient satisfaction and negatively affects postoperative outcomes [17]. Multimodal analgesia is one of the cornerstones of accelerated ERAS [18]. However, according to The PROSPECT (PROcedure-SPECifc Postoperative Pain Management) study group, opioids continue to be the most commonly used agents for pain management in the postoperative period due to the lack of specific recommendations for this surgery [19]. Opioid agents frequently used in postoperative pain management cause side effects such as opioid-related hyperalgesia, risk of opioid dependence, respiratory depression, nausea, and vomiting [4]. The main nonopioid agents of which postoperative analgesia efficacy has been investigated in LD to reduce these side effects are paracetamol, nonsteroidal antiinflammatory drugs, dexamethasone, gabapentinoids, ketamine, and DEX [15,20–22].

Dexmedetomidine is an antinociceptive agent that selectively stimulates α2 receptors at spinal and supraspinal levels [7]. Previous studies conducted in many different surgical groups have investigated the effects of DEX on postoperative pain intensity and/or opioid consumption with different doses and application methods (loading and/or infusion) [8,9,12,15,16]. Dexmedetomidine has been shown to have an analgesic effect in many studies [8,9,15,16]. However, there are also clinical studies with different results on this subject [10–12,14].

Zhang et al. [16] showed that DEX administered with an infusion rate at 0.5 μg/kg/h after a 1 μg/kg loading dose in LD caused a decrease in pain scores in the intraoperative period. Although the dose used in our study is similar, we think that the reason for these contradictory results may be the shorter duration of surgery (67 ± 12 min) in the mentioned study and the less tissue damage that occurred because the patients underwent surgery with the percutaneous endoscopic surgery method. In addition, the total dose of 5–10 mL 1% lidocaine, which was applied as infiltration when the intraoperative VAS score was >3, was not noted in this study. In addition, no difference was noted in terms of pain scores between the groups at the 24^th^ h postoperatively in the study. In another study, percutaneous LD and laminotomy were performed with conscious sedation. This study reported that infusion of DEX at a dose of 0.5 μg/kg/h after 0.5 μg/kg loading dose reduced need for fentanyl (24-h total consumption: Mean difference: −69.3 μg; 95% CI: −114.3 −24.4 μg; p = 0.003) but did not cause any improvement in pain scores [15]. In these studies, patients were operated on under conscious sedation with a percutaneous endoscopic method, unlike our patient group [15, 16]. Although LD can be performed with sedation + local anesthesia, spinal anesthesia, or epidural anesthesia [16,23,24], this procedure is frequently performed under GA in our clinic. The percutaneous endoscopic surgery method can provide benefits such as rapid neurological examination and effective use of the operating room. However, no clear evaluation criteria for preoperative anesthesia have been defined to predict which patient group is suitable for this surgical procedure. This method is often preferred in a limited patient population with low BMI, low ASA score, low Mallampati score, and good functional capacity [25]. In addition, patients undergo surgery in our clinic under GA due to concerns about the airway during sedation in the prone position, stress induced in awake patients, and the opportunity provided to the surgical team to work more comfortably during GA.

The analgesic efficacy of DEX in LD has also been investigated in nonintravenous applications. Alansary et al. [26] showed that 50 μg DEX added to epidural bupivacaine prolonged the time to first analgesic need and reduced the morphine requirement in the first 24 h compared to 50 μg fentanyl. There was no difference between the groups in terms of pain scores in the first 2 h, while scores were lower in the DEX group in the following hours. However, in this surgery, the drugs mentioned were administered through the epidural catheter both before the surgery and after skin closure. Deswal et al. [27] showed that the addition of 1 μg/kg DEX to ropivacaine applied as local infiltration before wound closure resulted in lower pain scores and fentanyl consumption in the postoperative period. In these studies showing the effectiveness of DEX, it was not applied systemically, and the drug was applied toward the end of the surgery. In our study, DEX infusion was discontinued when skin suturing and did not had a postoperative analgesic effect. There are also many studies in the literature indicating the analgesic effect of DEX infusion administered during the postoperative period [28,29].

Analgesia management during the intraoperative period may be a criterion determining the effectiveness of DEX. In the study conducted by Naik et al. [14] investigating the effectiveness of DEX in spinal surgery, the same dose of DEX was applied as in our study. This study, unlike our study, showed that opioid consumption decreased during the intraoperative period. However, the effectiveness of DEX was not shown in terms of opioid consumption and pain scores during the postoperative period. In our study, unlike this study, since nitrous oxide was used in the intraoperative period and paracetamol was used as a preemptive analgesic, no difference could be shown in terms of intraoperative fentanyl consumption. In addition, since long-acting intravenous methadone was used for analgesia in this study, we did not routinely administer opioids to the patients, thinking that the postoperative analgesic efficacy of DEX could be masked.

In our study, although postoperative fentanyl consumption was not statistically significant, it was higher in Group B during the postoperative period. We believe that it may be related to the short elimination half-life of dexmedetomidine (2.1–3.1 h) [30] and its administration only during the intraoperative period, as well as individual differences in pain sensitivity and tissue damage. Additionally, the minimal difference in fentanyl consumption between groups may have contributed to the lack of a statistically significant difference in NRS scores and postoperative complications.

The most common hemodynamic side effects during DEX infusion are bradycardia, hypotension, and hypertension. This situation is explained by the fact that DEX causes vasoconstriction, vasodilation, and reflex bradycardia due to presynaptic and postsynaptic α2-receptor activation [30]. However, these side effects are generally observed at higher infusion doses (>2 μg/kg/h) and may become more pronounced in patients at risk of atrioventricular block [31,32]. In our study, systolic blood pressure, mean blood pressure, heart rate, and atropine and noradrenaline needs during the intraoperative period and in the recovery unit were similar between the two groups. Since the patient with cardiac conduction abnormalities was not included in our study and the infusion dose was <2 μg/kg/h, hemodynamic side effects were not observed.

Postoperative nausea, vomiting, and shivering are important symptoms that negatively affect perioperative patient satisfaction. In meta-analyses published in recent years, it has been reported that DEX reduces these symptoms. Dexmedetomidine may reduce these opioid-related side effects through its effect of reducing opioid use [33–35]. Therefore, it was not surprising to see similar side effect rates between the two groups with similar opioid consumption in our study. In addition, the fact that all patients were prophylactically administered metoclopramide during the intraoperative period and granisetron was added to the pain pump during the postoperative period may be another reason for the similar nausea and vomiting rates. However, the similar complication rates observed between the two groups in our study may be due to the fact that DEX reduces these opioid-related side effects through its effect of reducing opioid use.

The limitations of this study are as follows: First, the study design was retrospective and had a heterogeneous patient population. In addition, the data of patients in whom DEX infusion continued throughout surgery were evaluated in this study. Since the data of patients in whom DEX infusion was discontinued for any reason were not included in the study, the hemodynamic side effects of DEX might not be fully revealed. Additionally, we excluded patients with uncontrolled hypertension because of the increased risk of hypotension, which could necessitate higher norepinephrine need, potentially influencing the hemodynamic parameters assessed as secondary outcomes in our study. Finally, the hemodynamic parameters after the DEX loading dose were not evaluated in the study due to incomplete recordings.

In conclusion, although many previous studies conducted in different types of surgery have indicated that DEX may have an analgesic effect, this effect was not shown in our study. This study revealed that intraoperative DEX administration in LMD under GA did not reduce opioid consumption and did not improve pain scores.

## Figures and Tables

**Table 1:** Demographic data.

		Total (n = 68)	Grup A (n = 34)	Grup B (n = 34)	P

		Mean ± SD	Mean ± SD	Mean ± SD	
Median (min–max)	Median (min–max)	Median (min–max)
Age		47.43 ± 11.96	49.06 ± 11.86	45.79 ± 12.01	0.264
		48.5 (27–65)	50 (27–65)	46 (28–65)	

Gender n (%)	Female	33 (48.53)	17 (50)	16 (47.06)	0.808

	Male	35 (51.47)	17 (50)	18 (52.94)	

Height (cm)		172.24 ± 9.83	170.5 ± 10.42	173.97 ± 9.01	0.147
		172.5 (151–197)	170 (151–197)	174 (160–192)	

Weight (kg)		77.91 ± 10.43	76.79 ± 11.12	79.03 ± 9.74	0.381
		80 (58–96)	74.5 (58–95)	80 (58–96)	

BMI		26.18 ± 2.52	26.3 ± 2.47	26.06 ± 2.60	0.700
		26.35 (20.2–29.7)	26.4 (20.2–29.7)	26.35 (21.2–29.7)	

BMI: Body mass index.

**Table 2:** Comorbidities, anesthesia and surgical parameters.

		Grup A (n = 34)		Grup B (n = 34)		P
		n	%	n	%	
Thyroid	No	30	(88.24)	33	(97.06)	0.356
	Yes	4	(11.76)	1	(2.94)	
Pulmonary disease	No	28	(82.35)	30	(88.24)	0.493
	Yes	6	(17.65)	4	(11.76)	
HT	No	25	(73.53)	27	(79.41)	0.567
	Yes	9	(26.47)	7	(20.59)	
DM	No	28	(82.35)	28	(82.35)	1.000
	Yes	6	(17.65)	6	(17.65)	
ASA score	1	8	(23.53)	9	(26.47)	0.779
	2	26	(76.47)	25	(73.53)	
Smoking status	No	18	(52.94)	15	(44.12)	0.467
	Yes	16	(47.06)	19	(55.88)	
Duration of anesthesia (min)		101.06 ± 21.13	100.5 (65–139)	94.65 ± 16.12	90 (65–140)	0.199
Duration of surgery (min)		72.53 ± 20.83	70 (40–115)	65.71 ± 15.60	61 (35–110)	0.247
Level	Single	29	(85.29)	31	(91.18)	0.709
	Two	5	(14.71)	3	(8.82)	
Bilateral surgery	No	25	(73.53)	29	(85.29)	0.230
	Yes	9	(26.47)	5	(14.71)	
Duration of extubation (min)		5.44 ± 3.92	5 (1–15)	5.35 ± 3.82	5 (1–15)	0.975

HT: Hypertension; DM: Diabetes mellitus, ASA: American Society of Anesthesiologists Physical Status Classification.

**Table 3:** İntraoperative hemodynamics.

	Grup A (n = 34)		Grup B (n = 34)		P
**Baseline**					
SBP	140.68 ± 14.06	143.5 (107–160)	143.82 ± 12.76	143.5 (111–160)	0.337
MBP	104.21 ± 11.78	105 (83–124)	105.35 ± 10.66	105.5 (84–131)	0.675
HR	76.79 ± 12.56	75.5 (53–99)	80.26 ± 13.28	78 (59–99)	0.272
**After surgical incision**					
SBP	111.41 ± 19.49	104.5 (86–160)	116.56 ± 17.75	114.5 (85–155)	0.259
MBP	85.68 ± 14.92	82 (65–120)	90.88 ± 15.00	90.5 (69–122)	0.156
HR	70.88 ± 11.05	70.5 (52–96)	74.59 ± 13.18	70.5 (57–100)	0.213
**Intraoperative 5** **^th^** ** min**					
SBP	110.29 ± 19.32	105.5 (84–160)	114.18 ± 17.80	114 (82–151)	0.392
MBP	81.24 ± 14.57	80 (63–120)	87.76 ± 14.82	90.5 (61–116)	0.072
HR	69.03 ± 10.14	67 (55–98)	69 ± 12.36	68.5 (51–100)	0.991
**Intraoperative 10** **^th^** ** min**					
SBP	108.85 ± 17.40	105.5 (79–140)	111.29 ± 17.40	110 (78–145)	0.565
MBP	81.29 ± 13.01	80.5 (61–107)	85.32 ± 15.23	81 (57–112)	0.245
HR	67.5 ± 10.13	66 (49–97)	66.24 ± 11.73	64 (51–98)	0.636
**Intraoperative 20** **^th^** ** min**					
SBP	109.74 ± 14.59	109 (74–144)	114.03 ± 16.77	113.5 (79–154)	0.264
MBP	82.41 ± 13.58	79 (57–109)	87.71 ± 16.51	86 (49–124)	0.153
HR	66.88 ± 8.35	66 (50–88)	68.15 ± 12.03	66 (53–100)	0.616
**Intraoperative 30** **^th^** ** min**					
SBP	108.79 ± 15.90	104.5 (80–146)	112 ± 14.10	111 (90–147)	0.382
MBP	80.38 ± 12.32	78 (55–105)	85.44 ± 12.01	84 (61–114)	0.091
HR	67.74 ± 8.61	66.5 (52–88)	68 ± 12.02	67 (53–100)	0.917
**Skin closure**					
SBP	118.29 ± 22.65	117.5 (85–169)	125.94 ± 15.36	124 (100–159)	0.108
MBP	88.41 ± 17.08	85 (65–134)	95.03 ± 13.00	94 (77–130)	0.077
HR	69.53 ± 11.76	67 (55–117)	71.56 ± 12.10	70 (52–99)	0.486
**Extubation**					
SBP	135.26 ± 18.94	133 (103–171)	136.44 ± 20.65	130 (94–169)	0.807
MBP	97.76 ± 17.48	96.5 (72–136)	103.88 ± 16.73	100.5 (66–149)	0.145
HR	75.94 ± 13.65	73.5 (54–113)	77.94 ± 13.63	77.5 (52–104)	0.548

SBP: Systolic blood pressure; MBP: Mean blood pressure; HR: Heart rate; Min: Minute.

**Table 4:** Hemodynamic parameters and Ramsey Sedation Scale in the recovery unit.

		Grup A (n = 34)		Grup B (n = 34)		p
**0** **^th^** ** min**						
SBP		126.15 ± 22.40	124.5 (91–176)	128.26 ± 19.30	128.5 (93–184)	0.678
MBP		90.06 ± 20.50	87 (62–141)	92.47 ± 18.45	92 (62–142)	0.612
HR		69.21 ± 13.53	67 (44–96)	67.35 ± 11.68	65 (50–100)	0.548
RSS	1	-	-	1	(2.94)	1.000
	2	24	(70.59)	24	(70.59)	
	3	10	(29.41)	9	(26.47)	
**5** **^th^** ** min**						
SBP		122.06 ± 20.49	122.5 (91–174)	128.12 ± 19.12	129 (80–169)	0.212
MBP		87.71 ± 13.91	83.5 (64–122)	93.35 ± 17.85	95 (61–136)	0.150
HR		67.32 ± 11.37	66.5 (50–95)	65.47 ± 11.61	63 (48–100)	0.508
RSS	1	-	-	1	(2.94)	0.734
	2	28	(82.35)	29	(85.29)	
	3	6	(17.65)	4	(11.76)	
**10** **^th^** ** min**						
SBP		123.41 ± 22.46	119 (88–180)	130.44 ± 16.22	132.5 (101–177)	0.144
MBP		87.88 ± 19.16	84.5 (50–133)	93.79 ± 14.10	93.5 (64–127)	0.152
HR		66.29 ± 11.32	64 (50–92)	65.79 ± 11.01	63 (50–99)	0.854
RSS	2	30	(88.24)	30	(88.24)	1.000
	3	4	(11.76)	4	(11.76)	
**20^th^** min						
SBP		124.35 ± 20.73	122.5 (90–170)	127.12 ± 19.29	126 (97–180)	0.571
MBP		92.71 ± 21.17	88.5 (62–147)	91.24 ± 15.94	94 (61–125)	0.747
HR		65.79 ± 10.29	64 (52–94)	64.74 ± 9.94	63 (52–92)	0.668
RSS	2	34	(100.00)	32	(94.12)	0.493
	3	-	-	2	(5.88)	
**30** **^th^** ** min**						
SBP		124.5 ± 20.23	123 (98–167)	128.5 ± 19.14	124.5 (100–177)	0.405
MBP		91.18 ± 15.02	90.5 (64–137)	92.56 ± 16.55	90 (62–129)	0.719
HR		65.18 ± 9.76	64 (52–92)	64.82 ± 9.40	62 (52–86)	0.880
RSS	2	34	(100.00)	34	(100.00)	-

SBP: Systolic blood pressure; MBP: Mean blood pressure; HR: Heart rate; RSS: Ramsey sedation scale; Min: Minute.

**Table 5:** Fentanyl consumptions and NRS scores.

		Grup A (n = 34)		Grup B (n = 34)		p
Fentanyl requirement in the recovery unit (μg)	No	21	(61.76)	16	(47.06)	0.223
	Yes	13	(38.24)	18	(52.94)	
Fentanyl consumption in the recovery unit (μg)		38.85 ± 6.18	35 (30–50)	41.67 ± 7.86	45 (30–60)	0.373
PO 2^nd^ h fentanyl consumption (μg)		57.65 ± 38.70	40 (0–180)	72.06 ± 38.91	60 (0–180)	0.052
PO 6^th^ h fentanyl consumption (μg)		111.76 ± 65.53	80 (20–260)	131.18 ± 69.49	120 (0–320)	0.148
PO 12^th^ h fentanyl consumption (μg)		173.53 ± 100,27	150 (40–460)	191.76 ± 89.86	190 (20–400)	0.290
PO 24^th^ h fentanyl consumption (μg)		251.76 ± 154.36	210 (60–700)	280 ± 125.82	260 (40–600)	0.156
Requirement for rescue fentanyl	No	27	(79.41)	26	(76.47)	0.770
	Yes	7	(20.59)	8	(23.53)	
The dose of rescue fentanyl (μg)		38.57 ± 6.90	35 (30–50)	36.25 ± 4.43	35 (30–45)	0.613
**NRS scores**						
2^nd^ h (at rest)		2.94 ± 1.41	3 (0–7)	2.82 ± 1.24	3 (0–5)	0.939
2^nd^ h (on moving)		4.65 ± 1.45	5 (0–9)	4.35 ± 1.25	5 (0–6)	0.397
6^th^ h (at rest)		2.59 ± 1.52	3 (0–8)	2.35 ± 1.04	3 (0–4)	0.688
6^th^ h (on moving)		4.18 ± 1.51	4 (0–9)	3.76 ± 1.28	4 (0–6)	0.258
12^th^ h (at rest)		2.03 ± 1.17	2 (0–6)	1.79 ± 1.12	1.5 (0–4)	0.429
12^th^ h (on moving)		3.5 ± 1.48	4 (0–8)	3.41 ± 1.46	3 (0–6)	0.845
24^th^ h (at rest)		2 ±1.41	2 (0–7)	1.74 ± 1.08	1.5 (0–4)	0.519
24^th^ h (on moving)		3.18 ± 1.55	3 (0–8)	3.26 ± 1.48	3 (0–6)	0.782

PO: Postoperative; μg: Microgram; NRS: Numerate rating scale.

**Table 6:** Perioperative drug requirements and patient outcomes.

		Grup A (n = 34)		Grup B (n = 34)		p
Time for MAS 9 (min)		15.44 ± 3.97	15 (10–30)	14.82±4.46	15 (10–25)	0.437
İO noradrenaline (n)	No	21	(61.76)	27	(79.41)	0.110
	Yes	13	(38.24)	7	(20.59)	
İO atropine (n)	No	28	(82.35)	28	(82.35)	1.000
	Yes	6	(17.65)	6	(17.65)	
Noradrenaline in the recovery unit (n)	No	31	(91.18)	33	(97.06)	0.614
	Yes	3	(8.82)	1	(2.94)	
Atropine in the recovery unit (n)	No	32	(94.12)	32	(94.12)	1.000
	Yes	2	(5.88)	2	(5.88)	
Patient satisfaction score		3.15 ± 0.56	3 (2–4)	3.38 ± 0.49	3 (3–4)	0.088
Length of hospital stay (day)		1.06 ± 0.24	1 (1–2)	1.15 ± 0.44	1 (1–3)	0.383

MAS: Modified Aldrete score; Min: Minute; IO: Intraoperative
